# Monitoring safety in a phase III real‐world effectiveness trial: use of novel methodology in the Salford Lung Study

**DOI:** 10.1002/pds.4118

**Published:** 2016-11-01

**Authors:** Sue Collier, Catherine Harvey, Jill Brewster, Nawar Diar Bakerly, Hanaa F. Elkhenini, Roxana Stanciu, Claire Williams, Jacqui Brereton, John P. New, John McCrae, Sheila McCorkindale, David Leather

**Affiliations:** ^1^ Respiratory Research and DevelopmentGlaxoSmithKline UK LtdUxbridgeUK; ^2^ Global Clinical Safety & Pharmacovigilance, Safety Evaluation and Risk ManagementGlaxoSmithKline UK LtdUxbridgeUK; ^3^ Respiratory Centre of ExcellenceGlaxoSmithKline UK LtdUxbridgeUK; ^4^ Salford Royal NHS Foundation TrustSalfordUK; ^5^ CK AspireChesterfieldUK; ^6^ Institute of Inflammation and Repair, Manchester Academic Health Science CentreUniversity of ManchesterManchesterUK; ^7^ NHS Salford Clinical Commissioning GroupUK and NIHR Clinical Research Network: Greater ManchesterUK; ^8^ Global Respiratory FranchiseGlaxoSmithKline UK LtdUxbridgeUK

**Keywords:** data linkage, electronic health records, pragmatic randomised controlled trial, real‐world evidence, safety reporting, Salford Lung Study, pharmacoepidemiology

## Abstract

**Background:**

The Salford Lung Study (SLS) programme, encompassing two phase III pragmatic randomised controlled trials, was designed to generate evidence on the effectiveness of a once‐daily treatment for asthma and chronic obstructive pulmonary disease in routine primary care using electronic health records.

**Objective:**

The objective of this study was to describe and discuss the safety monitoring methodology and the challenges associated with ensuring patient safety in the SLS. Refinements to safety monitoring processes and infrastructure are also discussed. The study results are outside the remit of this paper. The results of the COPD study were published recently and a more in‐depth exploration of the safety results will be the subject of future publications.

**Achievements:**

The SLS used a linked database system to capture relevant data from primary care practices in Salford and South Manchester, two university hospitals and other national databases. Patient data were collated and analysed to create daily summaries that were used to alert a specialist safety team to potential safety events. Clinical research teams at participating general practitioner sites and pharmacies also captured safety events during routine consultations. Confidence in the safety monitoring processes over time allowed the methodology to be refined and streamlined without compromising patient safety or the timely collection of data. The information technology infrastructure also allowed additional details of safety information to be collected.

**Conclusion:**

Integration of multiple data sources in the SLS may provide more comprehensive safety information than usually collected in standard randomised controlled trials. Application of the principles of safety monitoring methodology from the SLS could facilitate safety monitoring processes for future pragmatic randomised controlled trials and yield important complementary safety and effectiveness data. © 2016 The Authors Pharmacoepidemiology and Drug Safety Published by John Wiley & Sons Ltd.

## Introduction

### Study background and challenges

The Salford Lung Study (SLS) programme, encompassing two studies, is a pre‐licence pragmatic randomised controlled trial to evaluate the effectiveness and safety of a novel, once‐daily treatment for asthma^1^ and chronic obstructive pulmonary disease (COPD)^2^, ^3^ compared with existing maintenance therapy, in everyday clinical practice. Key differences between the SLS and standard randomised controlled trials (RCTs) have previously been described^1^, ^4^ (Table 1). With the objective of assessing an unlicenced treatment in a Phase III pragmatic randomised controlled trial conducted in the setting of routine primary care, the SLS study design necessitated the development of innovative methods to ensure patient safety and to meet regulatory requirements for safety reporting (Table 1). Since the SLS was initiated, the treatment under investigation, fluticasone furoate/vilanterol, has received a licence for the indications of asthma and COPD.^5^

**Table 1:** Challenges for safety monitoring and data collection in the Salford Lung Study compared with standard double‐blind randomised controlled trials

	Double‐blind RCT	SLS	Specific challenges to SLS
Patient characteristics	• Highly selected • Strict inclusion criteria, for example, spirometry • Multiple exclusions, for example, co‐morbidities • ‘Ideal’ patients	• Broad and heterogeneous population • Few exclusions • ‘Typical’ patients with multiple co‐morbidities	Large volume of complex serious adverse events expected; robust and timely safety monitoring as required for a randomised study of a pre‐licenced medication
Study conduct	• Treatment defined by protocol • Double blind, double dummy • Frequent review of patients often with multiple investigations over and above ‘usual care’ • Adherence monitored and encouraged • Study medicines provided by investigator	• Normal clinical care continues • ‘Usual treatment’ permitted • Minimal review • Medicines provided and collected in the usual way	Requires novel integrated methods for collecting safety data that do not interfere with patients' care nor change their usual HCP interactions
Outcomes	• Traditional clinical and scientific efficacy endpoints, for example, lung function, symptom scores, exacerbation • Safety	• Some traditional endpoints • Additional healthcare resource utilisation • PROs • Safety	

HCP, healthcare professional; PRO, patient‐reported outcomes; SLS, Salford Lung Study.

Patient safety is of paramount importance in clinical studies of investigational agents; however, minimal interaction with patients beyond routine clinical care is required if the collected data are to reflect real‐world experience. In the SLS, patients were enrolled through primary care practices using minimal exclusion criteria and without the extensive diagnostic testing typically used in RCTs.^1^, ^2^ This approach was designed to recruit a heterogeneous patient population that would be representative of asthma and COPD patients receiving treatment in routine primary care in the UK, including those with severe disease and multiple co‐morbidities; indeed, the strict eligibility criteria employed in typical registration studies means that patients with severe disease and co‐morbidities are frequently excluded. It was expected, therefore, that patients participating in the SLS would experience multiple serious adverse events (SAEs), thus generating a large volume of safety data, including higher rates of deaths, hospital admissions and new serious diagnoses, deterioration of chronic medical conditions and polypharmacy, than typically observed in traditional RCTs.

An innovative approach for identifying potential adverse events without interfering with patients' normal routines was essential for the SLS. This paper describes the novel methodology used to monitor patient safety, which enabled collection of real‐world evidence data.

### Pre‐study discussion with key stakeholders

In order to ensure the contribution of the SLS to the evidence base for regulatory and health technology assessments, the SLS study design team sought guidance on the study design under the joint scientific advice process from the National Institute for Health and Care Excellence and the Medicines and Healthcare Products Regulatory Agency. The meeting provided a first opportunity for regulators to assess the acceptability of our proposed safety monitoring processes, using routinely collected health data to alert the study team of possible adverse events. Additionally, the meeting allowed for an early discussion of the ethical equipoise of the study prior to submitting for ethical approval.

## Safety Monitoring and Data Collection Structure

### Information technology (IT) infrastructure

The NorthWest EHealth (NWEH) group created a bespoke IT infrastructure for the SLS, whereby potential safety events were captured through patients' electronic health records (EHRs), and in turn triggered clinical review by the specialist safety team.^6^ At recruitment, patients were invited to provide written informed consent allowing NWEH to access their entire medical record; EHRs from 80 general practitioner (GP) primary care practices in Salford and South Manchester, two secondary care university hospitals (Salford Royal National Health Service [NHS] Foundation Trust [SRFT] and the University Hospital of South Manchester NHS Foundation Trust [UHSM]), out of hours and accident and emergency (A&E) services and other national databases, were linked to create a linked database system (LDS).^2^, ^7^ The LDS is a fully validated software system^7^ (Supporting Information). Data were collected within the NHS firewall and complied with all NHS Information Governance standards.

**Figure 1:**
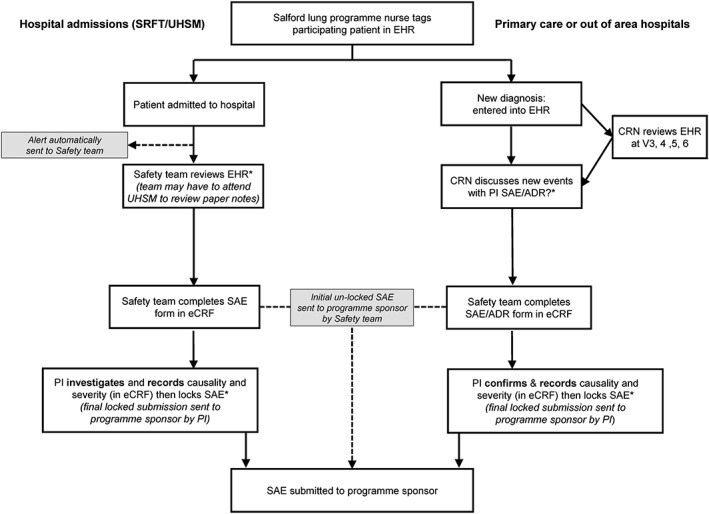
Chain of events following a potential serious adverse event trigger *Independent Clinical Research Associate monitoring to resolve and identify queries. ADR, adverse drug event; CRA, clinical research associate; CRN, clinical research nurse; eCRF, electronic case report form; EHR, electronic health record; PI, principal investigator; SAE, serious adverse event; SLS, Salford Lung Study; SRFT, Salford Royal NHS Foundation Trust; UHSM, University Hospital of South Manchester NHS Foundation Trust; V, visit.

Data from hospitals, GPs and out of hours services were refreshed daily, and amalgamated patient data were processed in a timely manner via the LDS, such that the clinical safety team were alerted each morning to any new potential safety events compared with the previous 24 h. Although the LDS captured, aggregated and extracted coded data from primary and secondary care EHRs, good clinical practice‐trained staff were required to review safety events along with non‐coded free text information, to determine if they met the criteria for an SAE as defined by the study protocol^1^, ^2^ (Figure 1). Upon determining an SAE, investigators or safety team staff completed SAE forms on behalf of the principal investigators (PIs), akin to the traditional safety monitoring processes in standard RCTs. The regulatory definitions of both SAEs and adverse drug reactions (ADRs) in the SLS have been described previously.^1^ All data feeds were monitored by NWEH, and contingencies for feed/system failures were established (Supporting Information).

The LDS system was configured to automatically alert when any pre‐defined ‘candidate events’ (potential SAEs) were coded in a patient's EHR. These pre‐coded alerts included the following: A&E visits and hospital admissions to SRFT or UHSM; events coded as ADRs in primary care EHRs; and potential sentinel events including liver events, renal events and neutropenia (see Table 2 for potential sentinel events). The safety team was not alerted to the following: outpatient visits to any hospital except UHSM that may have resulted in a new clinical diagnosis; A&E visits to any hospital other than UHSM and SRFT; and non‐coded (readable) events/diagnoses or letters entered in primary care EHRs. To ensure accurate capture of all events outside of SRFT and UHSM, the safety team received additional out of area data feeds on a monthly basis; although events detailed in these reports may have already been identified and reported by the primary care site, the feeds were reviewed to check for correct implementation of site safety monitoring procedures, ensuring that any missed events were promptly investigated.

**Table 2:** Sentinel events

Sentinel event	Trigger(s)
Torsade de Pointes and marked QTc prolongation	QTc > 550 msec, acquired long QT syndrome
Agranulocytosis	ANC < 500/μL
Anaphylaxis/anaphyloid reactions	Occurrence of
Hepatotoxicity	ALT >3 ULN; total bilirubin >2 ULN
Acute renal failure	1.5 × baseline increase in serum creatinine* OR ≥25% decrease in GFR compared with baseline
Seizures	Occurrence of
Stevens Johnson Syndrome	Occurrence of
Toxic epidermal necrolysis	Occurrence of

ANC, absolute neutrophil count; ALT, alanine aminotransferase; GFR, glomerular filtration rate; ULN, upper limit of normal.

*Baseline refers to last available recorded value; serum creatinine levels were not obtained at study entry.

### Safety monitoring visits

In the SLS, patients were recruited through their own general practices. Within each study site, one of the GPs acted as the PI and other GPs in the practice as sub‐investigators. Patients collected all their study medications from high‐street pharmacies that were also participating in the study. Study visits were kept to a minimum to reflect patients' routine clinical care and were scheduled as follows: Visit 1, patients were screened and offered the opportunity to participate and provide consent; Visit 2, baseline observations were conducted, and patients were randomised to study treatment; Visit 6 (12 months after Visit 2), final review of patients' EHRs. In addition, patients' EHRs were reviewed at Visit 3 (3 months), Visit 4 (6 months) and Visit 5 (9 months). Patients with COPD who had not been reviewed by their GP or practice nurse within the last 8 weeks received a telephone call to screen for potential safety events. Patients with asthma received these calls to assess their Asthma Control Test (ACT™) score, a primary endpoint of the study, as well as to screen for potential safety events.

### Structure of the safety monitoring teams

**Figure 2:**
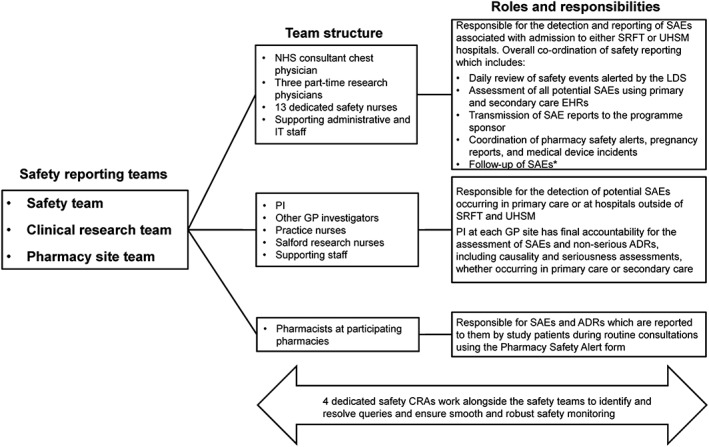
Structure and roles of the safety monitoring team *Follow‐up occurs at 7 days, 28 days and every 28 days thereafter until the event is resolved, stabilised or otherwise specified by the safety team. An additional earlier follow‐up, 48 h after the event, is implemented if the SAE is fatal or life threatening. Chest consultants, principal investigators, general practitioners and practice nurses have roles that are part of the NHS; research physicians have roles as part of NorthWest EHealth; pharmacists are independent staff; all other roles are employees of, or are funded by, the study sponsor. DR, adverse drug reaction; CRA, clinical research associate; EHR, electronic health record; GP, general practitioner; IT, information technology; LDS, linked database system; NHS, National Health Service; PI, principal investigator; SAE, serious adverse event; SLS, Salford Lung Study; SRFT, Salford Royal NHS Foundation Trust; UHSM, University Hospital of South Manchester NHS Foundation Trust.

In the SLS, as in other clinical trials, the PIs held overall responsibility for the detection and reporting of SAEs and ADRs. Safety monitoring and reporting was also supported by three teams: (i) the specialist safety team; (ii) the community clinical research team (CRT); and (iii) the pharmacy investigator site team, who were all trained in GCP and familiar with the study protocol (Figure 2).

The specialist safety team was responsible for the detection of SAEs associated with admissions to the SRFT and UHSM hospitals (accounting for most of the reported SAEs in the study), as well as for the overall coordination of safety monitoring in the SLS. An SAE was defined, using the ICH definition, as any untoward medical occurrence that (i) results in death, (ii) is life threatening, (iii) requires hospitalisation or prolongation of hospitalisation or (iv) results in disability/incapacity or in a congenital anomaly/birth defect. Other important adverse events could also be reported if in the judgement of the PI; they constituted SAEs. Any potential SAEs detected by the safety team that did not involve hospitalisation were referred to the PI for evaluation using a Safety Alert Form. The team's research physicians had delegated responsibility from the PI at each GP site and acted as sub‐investigators, to preliminarily assess relevant SAEs and non‐serious ADRs received from non‐GP sources such as hospital admissions. These preliminary assessments included an initial attribution of causality and severity determination according to the standard regulatory criteria described in the protocol, although the PI had final responsibility for these decisions (Figure 1).

The CRT at each GP investigator site was responsible (on behalf of the PI at each site) for the detection of potential SAEs occurring in primary care, which were identified through correspondence and/or consultations between patients and healthcare professionals at GP sites and hospitals/clinics other than SRFT and UHSM (Figure 2). Potential SAEs detected by the CRT were reported into electronic case report forms (eCRFs) with the support of the safety team at GP sites. Potential SAEs occurring in the community setting were also detected during the three monthly reviews of patients' EHRs, performed by research nurses. These events were reviewed by the PI at each study site, and any confirmed SAEs were submitted by the on‐site safety team to the study sponsor.

Study pharmacists were responsible for informing the safety team, via the Pharmacy Safety Alert form, of SAEs and ADRs that were self‐reported by patients during routine consultations.

### Processing of SAE data by the study sponsor

Possible and confirmed SAEs were reported to the study sponsor by both healthcare professionals and investigators. All SAEs were reviewed individually in near real time and also as quarterly aggregated data throughout the study by the sponsor's specialist safety teams. Any identified trends or emerging signals from any source were reviewed in near real time against the data documented in the sponsor's safety database. All SAE and safety data were reported by the sponsor to relevant regulatory authorities and investigators, as required by local and international regulatory reporting criteria.

### Refining the process

The methodology developed for safety monitoring in the SLS was novel, innovative, and designed to be as robust as possible, with several failsafe measures in place, that is, multiple data sources, a daily alert system, PIs supported by teams of safety physicians, research nurses and pharmacists and timely periodic reviews of EHRs. As the SLS progressed, the study team developed increased confidence in the safety monitoring and data collection processes, which allowed refinements to be made without compromising the principles of safety monitoring and reporting. The procedures for managing amendments to the safety monitoring process included raising and exploring ideas, planning, implementation, testing, review and the subsequent live application; these steps involved all members of the study team who would be impacted by the proposed changes, as well as the study Medical Governance Board and the sponsor's Clinical Safety Department. Examples of refinements implemented in the SLS safety monitoring and data collection processes are provided below:

1. Frequency of safety alerts for hospitalised patients

During the SLS, some patients had prolonged and medically complex SAEs. This resulted in multiple rounds of amendments to SAE reports, culminating in large, data‐dense records (Supporting Information). To reduce the frequency of alerts arising from minor updates to information already held in a patient's EHR, a change to the data collection process was implemented whereby the clinical safety team only received daily alerts for hospitalised patients at the time of hospital admission, on Days 7 and 28 post‐admission and on discharge from hospital, or death. This change facilitated streamlining of the safety monitoring and data collection process by reducing the number of required updates to SAE forms and hence the overall volume of data documented on the SAE forms, without compromising on timely reporting or data quality.

Completion of SAE reports

Initially, all SAE reports were completed by the safety team physicians; however, as more patients were accrued to the study, task allocation was refined to improve the overall efficiency of SAE reporting. All team members were trained to complete SAE reports, allowing physicians to focus on determining the causality and severity of SAEs and assessing the quality of SAE reports. Further streamlining included reorganising the safety team nurses, such that each nurse supported several GP and secondary care sites and was involved from the point of initiation to resolution of SAEs. Prior to this, nurses were divided into secondary care and GP site teams, resulting in multiple team members contributing to each SAE report. A communication log was integrated into the LDS to facilitate efficient sharing of information and safety decisions among safety team members.

In addition, because of the complexity of SAEs documented during the SLS, a dedicated team of specialist safety clinical research associates was trained to work alongside the safety team to ensure a smooth and robust monitoring process for SAE and ADR reporting (Figures 1 and 2).

Changes to IT systems

In order to fulfil the sponsor's post‐authorisation regulatory requirements while the SLS was still ongoing, the study was adapted and further refined to include an evaluation of the incidence of pneumonia events. To capture these data, a dedicated pneumonia form was added to the eCRF. In addition, the study sponsor changed its global pharmacovigilance database, which required associated changes to the IT infrastructure and to the GP systems to ensure continued robust and timely safety monitoring and data collection.

Expansion of the study footprint

Patients were initially recruited to the SLS in the Salford area of Greater Manchester, with most being admitted to the SRFT hospital for acute care. Recruitment was later expanded to areas in South Manchester, Trafford and Stockport, and it was deemed necessary to develop a safety alerting system in UHSM. In contrast to the EHRs held at SRFT, initially the safety team could only review and assess potential sentinel events and pneumonias associated with admissions to UHSM by physically accessing paper medical records held within the hospital, a situation that was comparatively more laborious and time‐consuming. Not all geographical areas have integrated EHR systems as in Salford, and hence, determining the key elements of secondary care required for safety monitoring allowed for a wider use of the methodology. However, the SLS study team was able to work with the existing systems in these areas and infer the elements required to monitor patient safety and collect safety data using the SLS methodology.

All changes to the SLS safety monitoring and data collection processes were audited by NWEH in conjunction with the Clinical Safety Department of the study sponsor to ensure that the quality of safety monitoring and data collection was maintained.

With these refinements, the SLS collected, processed, assessed and reported an average of 700 safety alerts per month, derived from a total of 7037 patients (*n* = 4237, asthma study; *n* = 2800, COPD study), at the time of writing. Full safety results will be published separately.

## Discussion

Monitoring of patient safety is an essential component of all clinical studies, including those conducted in the real‐world setting and in particular for those evaluating investigational therapies. To date, large, prospective, interventional randomised studies using EHRs to monitor patient safety in the pre‐licence setting are uncommon, and we believe that the SLS is the first of its kind. The primary analysis of SLS data found that once‐daily treatment with fluticasone furoate and vilanterol was associated with a lower rate of exacerbations than usual care, without a greater risk of serious adverse events, in patients with COPD and a history of exacerbations.^3^ Contrary to traditional RCTs, no relevant pre‐existing model for safety data collection was available at the time the SLS was designed. Hence, the methodology for safety data collection and reporting in the SLS had to be developed from scratch but was progressively refined and streamlined over the course of the study. The use of EHRs for safety monitoring in the SLS enabled safety data to be processed in near real time, a key advantage over paper‐based systems. Furthermore, the integration of multiple healthcare data sources facilitated the collection of more comprehensive safety data than typically captured on eCRFs in standard RCTs. We therefore propose that the methodology for collection and reporting of safety data in the SLS was as robust, if not more so, than the safety data collection processes employed in traditional RCTs.

Once SAEs and ADRs were reported to the study sponsor, usual clinical study procedures were followed in the SLS, similar to standard RCTs. However, a key difference in the SLS was that the safety teams monitored the evolution of safety events over time, that is, initial diagnoses were either confirmed or changed when further information became available. This resulted in a highly variable workload for the SLS safety team and for the Clinical Safety Department of the study sponsor and Risk Management team, as safety events had to be processed in near real time. It remains to be determined whether an actual increase in work volume and data processing time was incurred by the safety monitoring teams and sponsor.

The safety data collection and reporting processes of the SLS were refined over time to meet the requirements of the study, highlighting the flexibility of the methodology. Changes to requirements for safety data collection, such as the requirement to collect additional information on SAEs of pneumonia, were successfully accommodated by the system. We propose that the frequency and complexity of safety data collection can be adapted to suit different treatments, disease indications and drug development phases; for example, daily safety alerts may be required for a Phase II real‐world study or for a novel, investigational treatment, whereas less frequent safety alerts might be more appropriate in the post‐authorisation scenario. The expansion of the trial recruitment boundaries and subsequent incorporation of the UHSM EHRs into the safety monitoring and reporting system indicates that the methodology can be utilised across different geographical areas.

The growing use of IT systems in healthcare provides a considerable untapped resource for the monitoring and capturing of safety data for clinical trials. The development of methods for robust safety monitoring and data collection, with minimal disruption to routine clinical care (Table 1), could allow for a paradigm shift whereby clinical trials move from a predominantly hospital‐based setting to the community setting. In the future, the linked database system infrastructure and safety monitoring process exemplified here by the SLS could be adapted to cover larger geographical areas and integrate greater numbers and more extensive datasets, arguably providing better post‐marketing pharmacovigilance than conventional reliance on spontaneous event reporting. This methodology could also be applied to different disease areas and treatments with different safety profiles, to yield important complementary effectiveness and safety data.

## Conflict of Interest

N. D. B's employing organisation provides IT support to GlaxoSmithKline. He has received educational grants and speaker's fees from GlaxoSmithKline and Novartis and support for attending educational conferences from Boehringer Ingelheim, GlaxoSmithKline and Novartis. H. F. E. is employed by NorthWest EHealth on a grant from GlaxoSmithKline for the Salford Lung Study. J. P. N. is employed by NorthWest EHealth in part by a grant from GlaxoSmithKline for the Salford Lung Study; J. McC. is employed by NorthWest EHealth in part by a grant from GlaxoSmithKline for the Salford Lung Study. J. B. has received consulting fees from GlaxoSmithKline. She is employed by CK Aspire on a grant from GlaxoSmithKline for the Salford Lung Study. R. S. received educational funding from GlaxoSmithKline for a separate observational research study registered with the University of Manchester. She was employed by Salford Royal NHS Foundation Trust on a grant from GlaxoSmithKline for the Salford Lung Study. C. W. has been working as a complementary worker on behalf of GlaxoSmithKline for the Salford Lung Study. S. McC. has received consulting fees from GlaxoSmithKline. She is employed by the NHS Salford Clinical Commissioning Group as the diabetes and kidney clinical lead and by the National Institute for Health Research (NIHR) as the primary care lead for the NIHR Greater Manchester Clinical Research Network. S. C., C. H., J. B. and D. L. are employees of, and hold shares/stock options in, GlaxoSmithKline.

**Key Points**

- The Salford Lung Study programme, encompassing two studies, one in asthma and one chronic obstructive pulmonary disease, was the first prospective, randomised controlled trial to be conducted using electronic healthcare records to monitor patient safety.
- Integration of information from numerous healthcare data sources is likely to provide more comprehensive safety information than is usually collected in standard randomised controlled trials.
- Safety events were processed in near real time, and the linked database system created daily summaries that alerted a specialist safety team to potential safety events.
- Confidence in the initial validation process allowed the safety monitoring process to be refined and streamlined during the course of the study. The linked database system infrastructure could be used to monitor safety in different disease therapy areas, as well as different geographical locations, to yield important complementary effectiveness and safety data.

## Ethics Statement

Details of the study ethics have been reported.^3^

## Author Contributions

S. Collier is head of medical operations and medical monitor for the Salford Lung Study. She led the Salford Lung Study team to develop and implement improvements to safety monitoring processes. She developed the idea for the paper and led the authoring team, initial authoring and review. C. Harvey reviewed all safety data in‐stream and adapted the protocol for collection of additional details and analysis of events of pneumonia. She was responsible for the interpretation of all SAE safety data in the context of what is currently known (core safety information and SmPC). J. Brewster was involved in developing safety processes and in collection and review of SAE data into the safety database. N. D. Bakerly led the clinical safety team, and was involved in setting up the design of the Salford Lung Study, developing the clinical safety alerts, the initial discussion about the paper and subsequent review. H. F. Elkhenini was the safety physician in the studies. She was involved in developing the clinical safety alerts, safety monitoring process, testing of the system, discussion about the paper, writing and review. R. Stanciu participated in developing the collection form for pneumonia and the testing of the alerts system in secondary care. She contributed to writing and review. C. Williams was involved in streamlining and improving safety processes, including the creation of the safety alert form and new ways of working for the safety nurse team. J. Brereton led the implementation of primary and secondary care safety process and supported the transition of both primary and secondary sites. J. P. New led the development of the safety monitoring process for NWEH and was involved in setting up the design of the Salford Lung Study, the initial writing of paper and subsequent review. J. McCrae led the implementation of the IT infrastructure in the Salford Lung Study and was involved in the initial writing of paper and subsequent review. S. McCorkindale, as the GP representative, was involved in discussions about the development of safety alerts and initial discussion about the paper and review. D. Leather conceived the concept for the trial and was involved in setting up the Salford Lung Study and approval of the paper.

## Supporting information

Supplementary information
