# Supplementary material for: Monitoring safety in a phase III real‐world effectiveness trial: use of novel methodology in the Salford Lung Study
Source: Pharmacoepidemiol Drug Saf. 2016 Nov 1;26(3):344–52. doi: 10.1002/pds.4118 (PMC5347861; doi:10.1002/pds.4118)
Supplement: Supplementary file 1 — Supplementary information [file PDS-26-344-s001.docx]

**Supplementary information**

*Contingencies for feed/system failures*

The two most common causes of GP feed/system failures were the GP server being switched off and upgrades to the EHRs. If a feed was interrupted or delayed, the Clinical Trial Administrator (CTA) contacted the surgery to investigate the cause. Most issues were resolved within 48 hours, but if a feed was delayed by more than 5 days, a research nurse manually examined patient notes every 3 days to ensure that safety monitoring was appropriately maintained.

Hospital feed/system failures were much less common, as hospital EHRs are actively managed and EHRs are required 24/7 for clinical care. In the event of a data feed loss, the failure was managed by NWEH in a similar way to the above. An example of a feed failure is an upgrade to hospital EHRs, resulting in data being moved to a new server location and preventing simultaneous updates to the data feed; data feed was restored within 48 hours with the contingency plan.

*Testing the IT infrastructure*

To ensure reliable and accurate capture of safety events by the safety monitoring IT infrastructure, a number of internal validation tests were performed. A profile of a mock patient with safety-related events was created and the associated data were tracked through the IT system. Further, an audit of several patients’ EHRs was conducted over the course of a week to 10 days to ensure that all data were being extracted appropriately by the LDS, and data were compared to a manual data collection. The LDS is a fully validated software system, meaning that every step has been identified, documented and tested.

*Refining the process*

Here we provide further detail for example (1) of refinements implemented in the SLS safety monitoring and data collection processes.

During the SLS, some patients had prolonged and medically complex SAEs. With the initial daily safety alert system, SAE reports were updated in near real time, such that a patient’s diagnosis could have changed repeatedly over the course of an admission. For example, at the time of patient admission to hospital, the SAE report may have been initiated by the safety team with an entry of only symptoms (e.g. shortness of breath), for which there could be multiple potential diagnoses documented in the EHR (e.g. chest infection, exacerbation, heart failure). Only after further investigation and review of the results by the physician, could a definitive diagnosis be made. Consequently, SAE event terms could have changed frequently, adding to increased workload across the SLS. The volume of SAE data could thus have exceeded that available in the SAE reporting form without any clear benefit in outcomes, hence, the rationale for refinement to the process.
